# The *in vitro* assessment of the degree of monomer conversion, biaxial flexural strength, and mineral precipitation on demineralised dentine of novel resin composite containing monocalcium phosphate monohydrate and polylysine

**DOI:** 10.2340/biid.v12.44551

**Published:** 2025-08-20

**Authors:** Munchuporn Pariwatanasak, Saowapa Chadarat, Wisitsin Potiprapanpong, Sukanya Kyopun, Anne M. Young, Piyaphong Panpisut

**Affiliations:** a Faculty of Dentistry, Thammasat University, Pathum Thani, Thailand; b Department of Biomaterials and Tissue Engineering, UCL Eastman Dental Institute, Royal Free Hospital, Rowland Hill Street, London, UK; c School of Dentistry, Mae Fah Luang University, Chiang Rai, Thailand; d Thammasat University Research Unit in Dental and Bone Substitute Biomaterials, Thammasat University, Pathum Thani, Thailand

**Keywords:** Resin composite, biaxial flexural strength, monocalcium phosphate monohydrate, polylysine, degree of monomer conversion, remineralisation

## Abstract

**Objective:**

The development of ion-releasing resin composites is expected to reduce the risk of secondary caries. This study compared the degree of monomer conversion, biaxial flexural strength/modulus, elemental release, and remineralisation potential of a novel ion-releasing dental composite (Renewal MI) containing monocalcium phosphate monohydrate and polylysine.

**Materials and methods:**

The degree of monomer conversion after light curing for 20 s was determined (*n* = 8). The biaxial flexural strength and modulus after immersion in water for 24 h (*n* = 8) were evaluated. Additionally, the release of Ca and P after immersion in water for 2 weeks was assessed (*n* = 3). A disc specimen of the material (*n* = 1) was attached to the demineralised dentine and then immersed in simulated body fluid for 2 weeks to qualitatively determine mineral precipitation on dentine. The commercial comparison included Filtek Z350 XT, EQUIA Forte HT, FUJI VII, and FUJI II LC.

**Results:**

FUJI II LC demonstrated the highest degree of conversion (97.6%) compared to Renewal MI (57.2%) and Filtek Z350 XT (61.2%). The highest flexural strength was observed in Filtek Z350 XT (271 MPa), followed by MI (135 MPa), FUJI II LC (109 MPa), EQUIA Forte HT (50 MPa), and FUJI VII (35 MPa). The biaxial flexural modulus of Renewal MI (3.2 GPa) was comparable to that of EQUIA FORTE HT (3.8 GPa) and FUJI II LC (3.6 GPa). Ca and P release of MI (11 ppm, 45 ppm) was higher than that of FUJI VII (<0.1 ppm, 0.7 ppm). The precipitation of mineral precipitates in dentinal tubules of demineralised dentine was not detected in all materials.

**Conclusion:**

Renewal MI demonstrated a degree of conversion similar to commercial resin composite but exhibited lower strength. However, its strength was much higher than conventional glass ionomer cements. The material promoted the high release of elements, which was expected to encourage the remineralising actions.

**KEY MESSAGES**

1. The novel composite, containing monocalcium phosphate and polylysine (Renewal MI), demonstrated lower polymerisation compared to resin-modified glass ionomer cement (GIC), but was similar to commercial resin composite.
2. Renewal MI demonstrated comparable strength to resin-modified GIC (FUJI II LC) and greater strength than conventional GIC (FUJI VII), with the added benefit of releasing essential elements, such as calcium (Ca) and phosphorus (P).

## Introduction

Secondary caries is a common cause of resin composite restoration failure. This may be due to the tendency of resin composites to accumulate dental biofilms and bacterial penetration in gaps between the tooth and the restoration [1, 2]. A study indicated that the most frequent occurrence is in posterior restorations and at the gingival margin [3]. Treatment options typically involve replacement (72%), which can result in further loss of sound tooth structure and weakening of the restored tooth [3]. The direct costs for caries management in the age group of 12–65 years globally were estimated to be between 10.2 and 36.2 billion USD [4]. It was reported that 5.7% of restorations required replacement during an observation period of 2.8 years [5], leading to increased healthcare expenditure. The replacement of restoration also accounted for 32.5% of restorative works [6]. This replacement procedure is also associated with increased pollution and waste generated from the dental procedure [7].

Glass ionomer cements (GICs) may exhibit higher anti-caries benefits compared to resin composites, which are expected to aid in the prevention of secondary caries [8]. It has been reported that the longevity of GICs in posterior teeth placed in low caries risk patients was 100% at 10 years [9]. However, the study also indicated that the longevity of GICs varies, with mean failure rates ranging from 16% to 21% after 2 years [10]. It may be possible that the survival rate of GICs could be significantly lower in high caries-risk patients. It was reported that the median survival rate of GIC restorations in non-load-bearing applications, such as root caries restorations, was only ~3.57 years [11]. Another main concern with GICs is their lower mechanical strength than resin composites [12].

The limitation of resin composites is the lack of remineralising and antibacterial actions [13]. Recent studies introduced a new experimental resin composite containing monocalcium phosphate monohydrate (MCPM) and polylysine (PLS) called Renewal MI. This exhibited a new self-adhesion mechanism through micromechanical locking by forming exceptionally long resin tags (> 400 μm) in demineralised dentine [14]. Additionally, the composite can absorb water from tooth surfaces and enhance mineralisation through calcium and phosphate ion release, which promotes the formation of hydroxyapatite [15]. The current commercial ion-releasing resin composites generally exhibited lower mechanical strength and wear compared to commercial resin composite due to the absorption of water [12, 16]. The increase in surface roughness may additionally promote biofilm accumulation that could increase the risk of secondary caries [13]. The clinical performance at 2 years of Cention N also demonstrated significantly decrease in marginal integrity [17] which could potentially lead to risk of bacterial microleakage. However, it was suggested that Cention N release of multiple ions which may contribute to *in vitro* antibacterial effects [18].

Renewal MI was also expected to help reduce the risk of secondary caries due to its antibacterial action. The antibacterial effects of the material were primarily contributed by the addition of epsilon-polylysine. The U.S. Food and Drug Administration has approved polylysine for use as a food preservative in the manufacturing of canned foods. Polylysine is a cationic polymer that exhibits broad-spectrum antibacterial activity while exhibit minimal toxic effects to the human system [19]. The main mechanism of action of polylysine was reported to be the disruption of the bacterial membrane and cellular processes [20]. A high level of polylysine (10–20 wt%) was incorporated into resin composites to encourage the polylysine release, but it led to a significant reduction in strength [21]. The concentration of polylysine incorporated in the resin composite was therefore subsequently optimised in Renewal MI. It was demonstrated that Renewal MI exhibited a significant reduction in *S. mutans* biofilm formation without a detrimental reduction in the mechanical strength of the material [22].

This combination of remineralising and antibacterial effects is expected to make Renewal MI highly effective for treating caries-affected dentine in minimally invasive dentistry. The addition of hydrophilic components such as MCPM and polylysine may lead to a lower strength than the commonly used resin composite [21]. The reactive components may additionally affect the light penetration required for the polymerisation of resin composites [23].

The objective of this study was to compare the polymerisation and strength of a novel composite containing MCPM and polylysine (Renewal MI) with that of commercial resin composite and GICs. Furthermore, the ability of materials to release essential elements such as calcium (Ca) and phosphorus (P) was also evaluated. The null hypothesis was that Renewal MI would not demonstrate statistically significant differences in monomer conversion, biaxial flexural strength (BFS)/biaxial flexural modulus (BFM), and elemental release when compared to the other materials. The interaction between materials and demineralised dentine was also qualitatively determined.

## Materials and methods

### Materials

**Figure 1:**
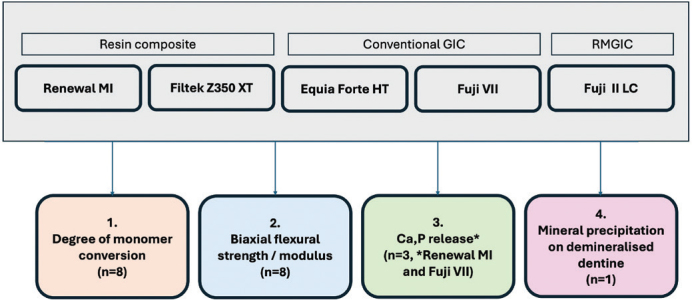
The protocol used in the current experiment.

The materials tested in the study are listed in Table 1. The resin composites included Renewal MI and Filtek Z350. The GICs were resin-modified GIC (RMGIC, FUJI II LC) and conventional GICs (EQUIA Forte HT and FUJI VII). The diagram for experimental design in the current study is provided in Figure 1.

**Table 1:** The materials used in the current study.

Materials	Type	Composition		Suppliers	Lot no.
		Liquid phase	Filler phase		
Renewal MI	Resin composite	UDMA, PPGDMA, 4-META, CQ	Barium alumino silicate, Monocalcium phosphate, Polylysine	Davis, Schottlander and Davis Dental Company, Letch-worth, UK	257053
Filtek Z350 XT	Resin composite	Bis-GMA, UDMA, other dimethacrylates	Zirconia/silica (0.6 µm)	3M ESPE, St. Paul, MN, USA	NE66690
Equia Forte HT	Conventional glass ionomer cement	polybasic carboxylic acid, polyacrylic acid, water	fluoroaluminosilicate glass, iron oxide	GC, Tokyo, Japan	2208171
Fuji II LC	Resin-modified glass ionomer cement	HEMA, polyacrylic acid, water	fluoroaluminosilicate glass	GC, Tokyo, Japan	2210244
Fuji VII	Conventional glass ionomer cement (containing calcium phosphate)	polyacrylic acid, polybasic carboxylic acid, polyacrylic acid	fluoroaluminosilicate glass, Recaldent or casein phosphopeptide-amorphous calcium phosphate (CPP-ACP)	GC, Tokyo, Japan	2212081

UDMA: Urethane Dimethacrylate; PPGDMA: Polypropylene Glycol Dimethacrylate; 4-META: 4-Methacryloxyethyl Trimellitic Anhydride; Bis-GMA: Bisphenol A-Glycidyl Methacrylate; HEMA: 2-Hydroxyethyl Methacrylate.

### Degree of monomer conversion

The degree of monomer conversion (DC) of the resin-based materials (RENEWAL MI, FILTEK Z350 XT, and FUJI II LC) was determined using attenuated total reflectance-Fourier transform infrared spectroscopy (ATR-FTIR, Nicolet iS5, Thermo Fisher Scientific, Waltham, MA, USA) (*n* = 8). Materials were prepared according to the manufacturer’s instructions and placed onto the ATR diamond. Each sample was confined using a standardised metal ring (10 mm internal diameter, 1 mm thickness) and covered with an acetate sheet to ensure a flat surface. Pre-polymerisation FTIR spectra were recorded. The samples were then light-cured for 20 s using an LED unit (Demi Plus, Kerr Dental, Brea, CA, USA), which delivered an irradiance of approximately 1,100–1,330 mW/cm² with a tip diameter of approximately 8.5 mm. Then, the FTIR spectra of the cured samples were immediately recorded. All spectroscopic measurements were performed from 400 to 4,000 cm^-1^ at 4 cm^-1^ resolution, averaging 8 scans per measurement. The test was performed at 25 ± 1^o^C. The DC was calculated using the following equation [24].

$$\text{DC}=\frac{100\left(\Delta {\text{A}}_{0}-\Delta {\text{A}}_{\text{t}}\right)}{\Delta {\text{A}}_{0}}$$Equation 1

where, ΔA_0_ and ΔA_t_ represent the peak heights of the methacrylate C–O stretching vibration (1,320 cm^-1^, measured from the background at 1,335 cm^-1^) before polymerisation and at time t (immediately after 20 s light-curing) [24, 25].

### Biaxial flexural strength and modulus

**Figure 2:**
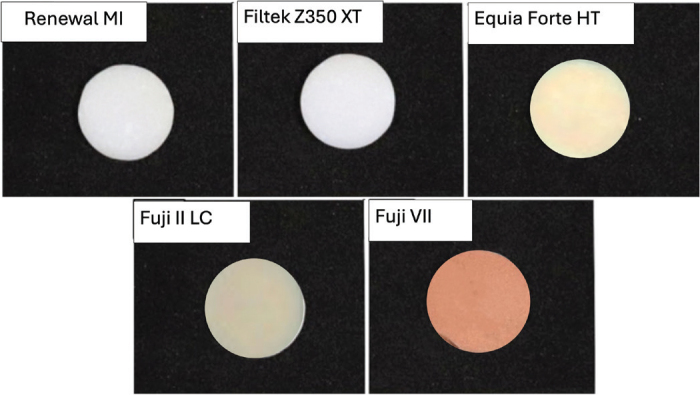
The appearance of each material after curing for 20 s on the top and bottom surfaces.

The BFS test was chosen because the test provided results with less variability than the three-point bending test [26]. Disc specimens (10 mm diameter, 1 mm thickness) were fabricated for each material group (*n* = 8). For resin-based materials (RENEWAL MI and FILTEK Z350 XT), the pastes were placed in metal rings and sandwiched between acetate sheets and glass slabs. Light curing was performed for 20 s with the same LED curing unit used for DC measurement on both top and bottom surfaces in a circular motion. For the RMGIC (FUJI II LC), the capsules were mixed in an amalgamator (CapMix, 3M, Saint Paul, MN, USA) for 10 s, then injected into the metal ring and light-cured following the same protocol as the resin composites. The conventional glass ionomers (EQUIA FORTE HT and FUJI VII) were prepared by mixing their capsules in the amalgamator for 10 s. Then, injecting them into metal rings and allowing them to self-cure without light activation. All specimens were maintained at room temperature for 24 h before being removed from the rings and immersed in 10 mL of artificial saliva at 37°C for an additional 24 h before testing. The appearance of specimens after curing for 20 s on the top and bottom surfaces of each material is provided in Figure 2.

For mechanical testing, specimen thickness was measured using a digital vernier calliper before positioning each disc on a ball-on-ring testing jig mounted in a universal testing machine (Shimadzu AGSX, Shimadzu, Tokyo, Japan). Tests were conducted using a 500 N load cell at a crosshead speed of 1 mm/min until specimen failure. The maximum load was recorded, and BFS and BFM were calculated using the following equations [24]:

$$\text{BFS}=\frac{\text{F}}{{\text{d}}^{2}}\left\{\left(1+\text{v}\right)\left[0.485\ln\left(\frac{\text{r}}{\text{d}}\right)+0.52\right]+0.48\right\}$$Equation 2

$$\text{BFM}=\left(\frac{\Delta \text{H}}{\Delta {\text{W}}_{\text{c}}}\right)\times \left(\frac{{\text{β}}_{\text{c}}{\text{d}}^{2}}{{\text{q}}^{3}}\right)$$Equation 3

where *F* represents failure load (*N*), *d* is specimen thickness (*m*), *r* is the radius of the testing jig’s circular support (*m*), and *v* represents Poisson’s ratio (0.3) [24]. $\frac{\Delta \text{H}}{\Delta {\text{W}}_{\text{c}}}$ denotes the slope of the force-displacement curve (N/m), while β_c_ (0.5024) and *q* represent the centre deflection function and the ratio of the support radius to the specimen radius, respectively.

The fracture surface of a representative specimen from each group was coated with gold using a sputter coater (Q150R, Quorum Technologies, East Sussex, UK). Fracture site analysis was performed using a scanning electron microscope (SEM, JSM 7800F, JEOL, Tokyo, Japan) equipped with energy-dispersive X-ray (EDX, X-Max 20, Oxford Instruments, Abingdon, UK). EDX mapping was performed on the fracture site of the specimen. The examination was conducted at an accelerated voltage of 15 kV.

### Elemental release (Ca, P)

The manufacturer reported that RENEWAL MI and FUJI VII were incorporated with calcium phosphate reactive fillers to enhance Ca and P release. Renewal MI includes MCPM [14], while Fuji VII contains Casein Phosphopeptide-Amorphous Calcium Phosphate (CPP-ACP) [27]. Hence, RENEWAL MI and FUJI VII were selected to determine the release of Ca and P in deionised water (*n* = 5). The disc-shaped samples (10 mm diameter, 1 mm thickness) were fabricated and immersed in 5 mL of deionised water. These specimens were stored at 37°C for a 4-week period. Then, elemental analysis of the storage solutions was conducted using inductively coupled plasma optical emission spectrometry (ICP-OES, Optima 8300, PerkinElmer, Waltham, MA, USA) [28]. The instrument was calibrated using an environmental standard containing 26 components (CPA Chem, Bogomilovo, Bulgaria). Measurements were performed at wavelengths of 317 nm for Ca and 213 nm for P, with detection ranges of 0.1–50 ppm and 0.5–20 ppm, respectively.

### SEM analysis of demineralised dentine

Five extracted human third molars of comparable size and without visible carious or non-carious lesions were collected from Thammasat University Hospital, Pathum Thani, Thailand. The study received ethical approval from the Human Research Ethics Committee of Thammasat University (Science), Thailand (COE number 025/2566). The ethics committee waived consent for tooth collection since patient identification was not required. The teeth were stored in a 0.1% thymol solution at room temperature for up to 3 months before testing.

**Figure 3:**
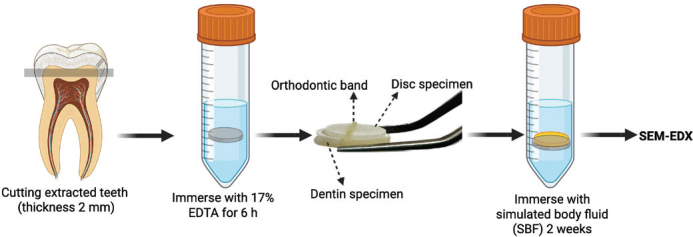
The schematic illustration of mineral precipitation assessment. Created in BioRender. Panpisut, P. (2025). https://BioRender.com/q26d152.

The teeth were cut horizontally to obtain tooth slices with a thickness of 2 ± 0.5 mm. The dentine specimens were demineralised in 1 mL of 17% ethylenediaminetetraacetic acid (EDTA, Faculty of Dentistry, Mahidol University, Bangkok, Thailand) at 37°C for 6 h [29]. Following this, the five specimens were rinsed with deionised water, and each specimen was attached to each of the five materials (disc specimens) using orthodontic elastic bands. The specimens were subsequently immersed in 10 mL of simulated body fluid (SBF) prepared according to ISO BS ISO 23317:2014. Implants for surgery: *In vitro* evaluation for apatite-forming ability of implant materials [30]. The tubes were incubated at 37°C for 2 weeks. After incubation, the specimens were dried by blotting. The material specimens were removed, and the dentine surface was coated with gold using a sputter coater and examined under SEM-EDX using the protocol described in the BFS test method above. This experiment is a qualitative assessment (Figure 3).

### Statistical analysis

The quantitative results (DC, BFS, BFM, and elemental release) reported in the current study were mean and standard deviation (SD). Data were statistically analysed using Prism 10.0 for macOS (GraphPad Software, San Diego, CA, USA). The normality of the data was examined using the Shapiro–Wilk test. One-way ANOVA followed by the Tukey HSD test. The significance value was set at *p* = 0.05. The sample size estimation was performed using G*Power version 3.1.9.6 (University of Dusseldorf, Germany) [31]. Results from the previous study [21] were used to estimate the sample size for each test to obtain a power greater than 0.95 at an alpha level of 0.05 for one-way ANOVA.

## Results

### Degree of monomer conversion

**Figure 4:**
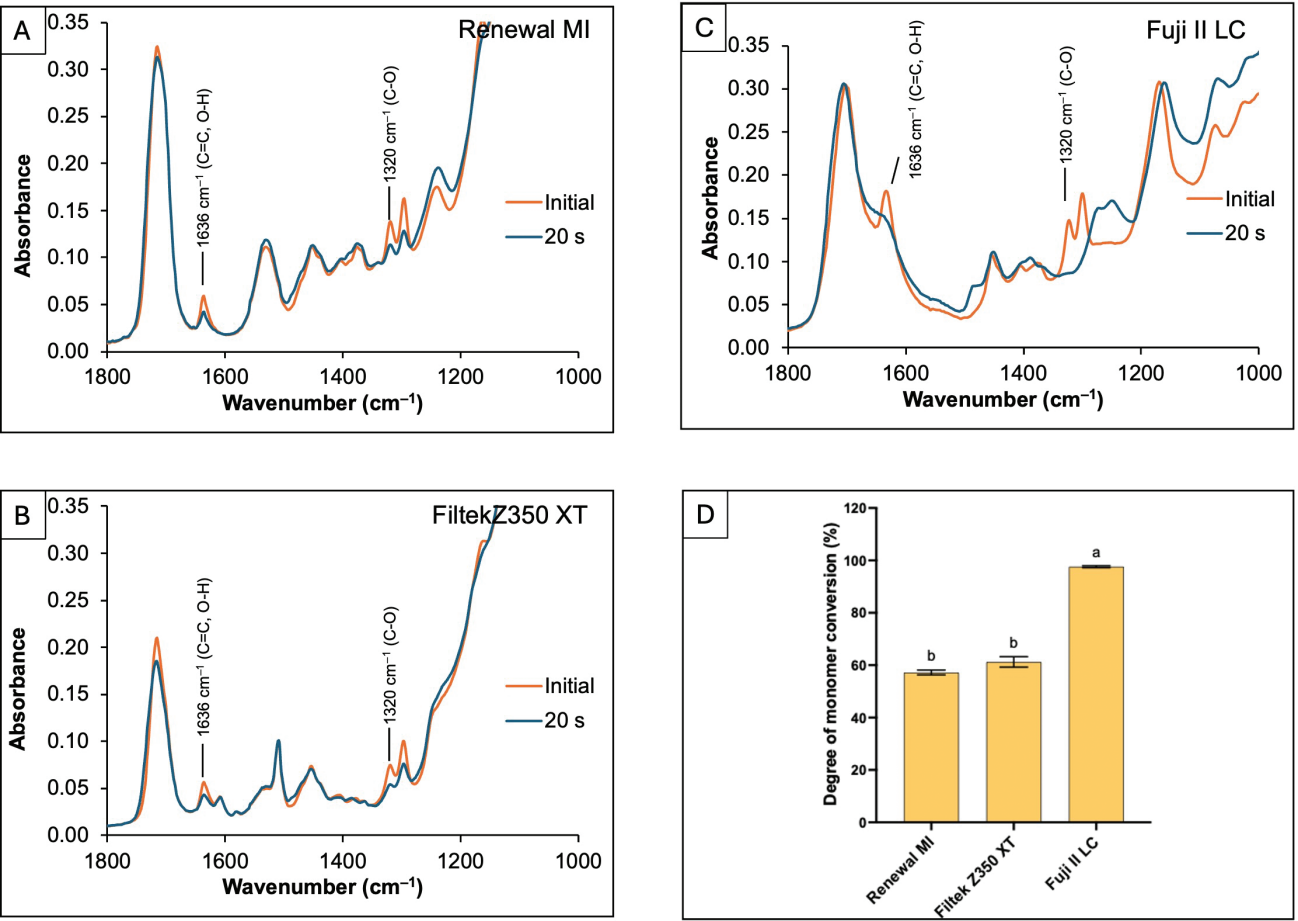
(A–C) The FTIR spectra of resin-based materials (Renewal MI, FILTEK Z350 XT, FUJI II LC, respectively) before and immediately after light-curing for 20 s. (D) The degree of monomer conversion of each material after light-curing for 20 s. Error bars are SD (*n* = 8).

The reduction of peaks representing the polymerisation of methacrylate groups after light-curing was observed in all materials (Figures 4A–C). A substantial reduction was detected with FUJI II LC. The degree of monomer conversion of FUJI II LC was calculated to be 97.6 ± 0.4%, which was significantly higher than that of RENEWAL MI (57.2 ± 1.1%) and FILTEK Z350 XT (61.2 ± 2.4%) (*p* < 0.05) (Figure 4D). However, the DC of RENEWAL MI was comparable to FILTEK Z350 XT (*p* = 0.1681).

### Biaxial flexural strength and biaxial flexural modulus

**Figure 5:**
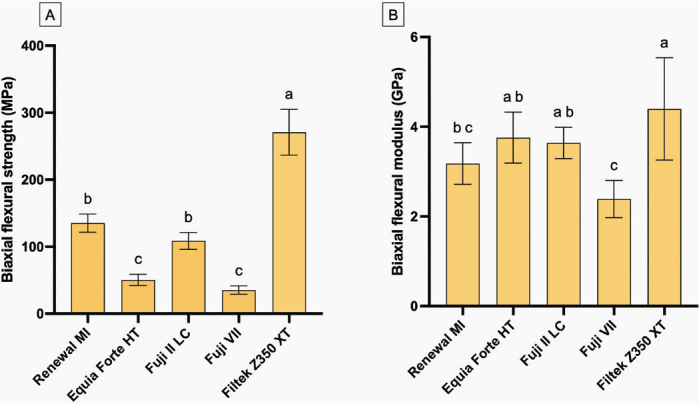
(A) The biaxial flexural strength (B) and modulus (C) after immersion in artificial saliva for 24 h. Error bars are SD (*n* = 8).

**Figure 6:**
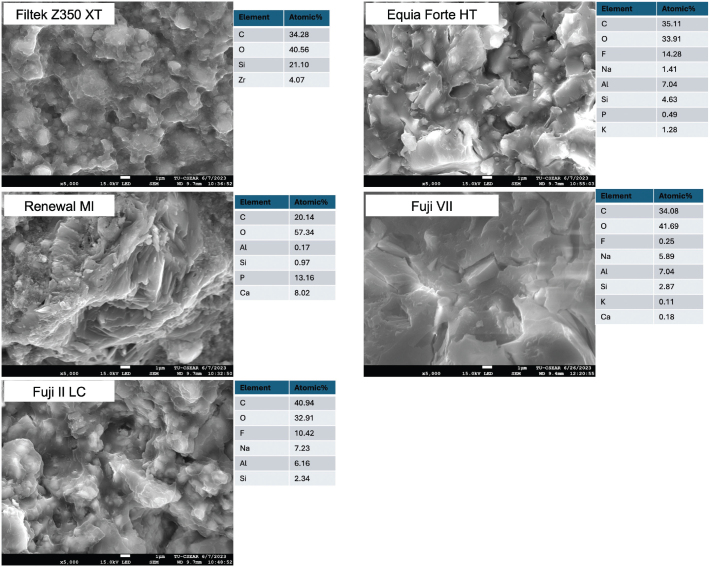
The SEM image of the fracture site of the representative specimen after BFS testing with the elemental composition from the EDX.

FILTEK Z350 XT exhibited the highest BFS (271 ± 41 MPa) in comparison to other materials (*p* < 0.05) (Figure 5A). The BFS of RENEWAL MI (135 ± 14 MPa) was similar to that of FUJI II LC (109 ± 13 MPa) (*p* = 0.10) but significantly higher than EQUIA FORTE HT (50 ± 9 MPa) (*p* < 0.01) and FUJI VII (35 ± 6 MPa) (*p* < 0.01). The BFS of EQUIA FORTE HT was similar to FUJI VII (*p* = 0.59). For BFM (Figure 5B), FILTEK Z350 XT showed the highest value (4.4 ± 1.4 GPa) compared to other materials. The BFM of RENEWAL MI (3.1 ± 0.6 GPa) was comparable to that of FUJI II LC (3.6 ± 0.4 GPa), EQUIA FORTE HT (3.8 ± 0.7 GPa), and FUJI VII (2.4 ± 0.5 GPa) (*p* > 0.05). The fracture surface analysis of the BFS-tested is provided in Figure 6. EDX mapping indicated that RENEWAL MI exhibited the highest proportion of calcium (Ca) and phosphorus (P) levels.

### Elemental release (Ca, P)

This study compared the release of Ca and P from the materials that contained additional calcium phosphate fillers, such as MCPM in RENEWAL MI and CPP-ACP in FUJI VII. The level of Ca release from FUJI VII was lower than the detection limit of the instrument. The cumulative release at 2 weeks of Ca obtained from RENEWAL MI was 11.1 ppm. For P release, the cumulative P release at 2 weeks of RENEWAL MI (44.7 ± 1.7 ppm) was significantly higher than that of FUJI VII (0.7 ± 0.0 ppm) (*p* < 0.01).

### SEM analysis of demineralised dentine

**Figure 7:**
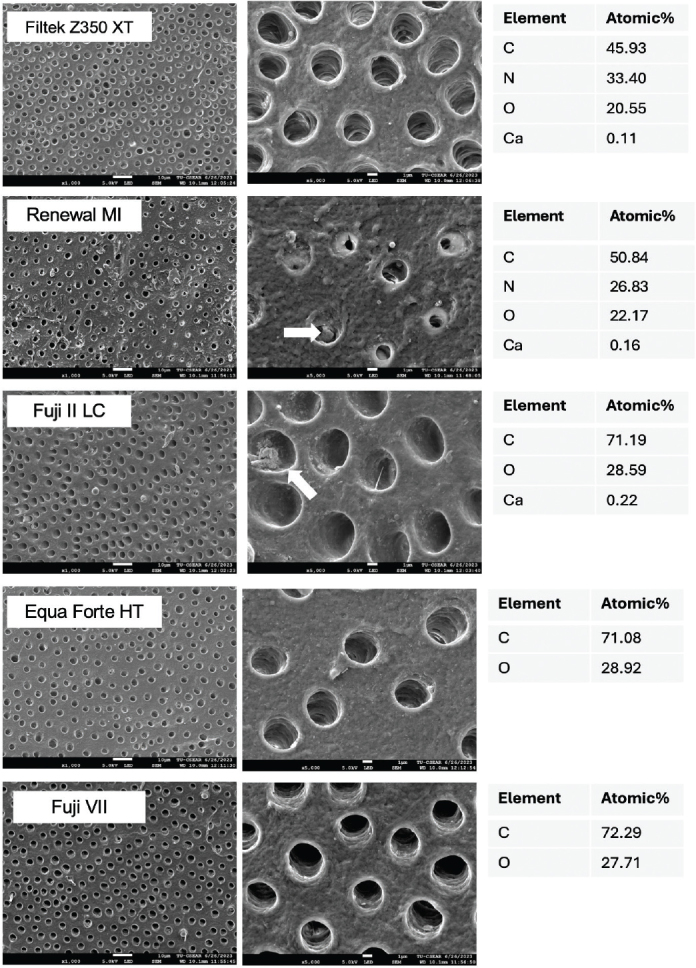
Dentine surface at low (x1,000) and high (x5,000) magnification after contact with disc specimens from each material for 2 weeks. Arrows indicated the possible mineral precipitation that filled dentinal tubules. EDX results were obtained by mapping the whole area from the low magnification (x1,000) of the SEM image.

The dentine surface exhibited minimal mineral precipitation within the dentinal tubules across all groups (Figure 7). However, some precipitates were observed on the dentine surface that was attached with RENEWAL MI and FUJI II LC. A lower level of Ca was detected within the dentine attached to FILTEK Z350 XT, and none was detected with EQUIA FORTE HT and FUJI VII.

### Discussion

This study compared the degree of conversion (DC), BFS/BFM, elemental release (Ca, P), and dentine remineralisation of resin composite containing MCPM and polylysine (Renewal MI) with commercial composite and GICs. The findings of the current study indicated that DC, BFS/BFM, and elemental release of Renewal MI were significantly different from those of the commercial materials. Hence, the null hypotheses were rejected.

The high DC of resin-based materials was expected to promote high mechanical properties [32], reduce the risk of monomer leaching [33], and ensure good colour stability [34] for the materials. The highest DC of resin-based materials in the current study was observed with FUJI II LC. This could be attributable to the use of HEMA as the primary methacrylate monomer in the material. The water may help plasticise the polyHEMA polymerising mixture and promote the movement of free radicals, thus enhancing polymerisation of RMGICs. This finding is also in accordance with the previous study, which reported that RMGICs demonstrated higher DC (73–88%) than other resin composite materials (50–59%) [12]. RENEWAL MI still exhibited a value within the range obtained from the commercial materials (40% to 75%) [35]. RENEWAL MI also demonstrated DC comparable to that of commercial ion-releasing resin composites, such as Cention N (Ivoclar Vivadent, Schaan, Liechtenstein) (59 ± 2%) [12]. It is important to note that the DC may continue to increase following the initial curing process through post-curing. A study reported that the DC was increased by approximately 47% after incubation at 37°C for 24 h [36].

RENEWAL MI exhibited lower strength than FILTEK Z350 XT, as was expected, which may be attributable to various factors. The addition of hydrophilic components to RENEWAL MI (MCPM and polylysine) was reported to enhance water sorption to a high level (32–56 μg/mm³) [15]. This may result in plasticisation of the polymer matrix and potential hydrolysis of bonds between the matrix and filler particles. The reactive fillers also increase water solubility, leading to porosity within the material after component release [15, 37]. The strength at 24 h of Renewal MI obtained in the current study (135 MPa) is comparable to that of similar materials reported in a previous study (121–141 MPa). This earlier study also indicated that the strength may be decreased to 73–101 MPa after 3–6 months when the hydrophilic components were increased [15]. Despite the lower strength compared to FILTEK Z350 XT, the strength of Renewal MI was still much higher than that of conventional GICs (35–50 MPa) and passed the requirement (80 MPa) of BS EN ISO 4049:2019: Dentistry-Polymer-based restorative materials [38]. The novel composite may serve as an alternative to GICs when high occlusal forces are a concern. However, long-term assessment of mechanical properties and comparisons with other ion-releasing materials should be conducted in future studies. The fracture surface analysis of the BFS-tested specimen revealed that FILTEK Z350 XT exhibited a smoother surface, likely due to its nanosized fillers (4–10 nm and 20 nm in clusters) [39].

The release of elements such as calcium (Ca) and phosphorus (P) was detected with MI, which was higher compared to FUJI VII. This could be attributed to the fact that the release of Ca and P depends on the solubility of calcium phosphate. A lower Ca/P ratio results in higher water solubility of the material [40]. The main calcium phosphate component of Renewal MI is MCPM, which has a low Ca/P ratio (0.5) [41]. This may allow rapid dissolution of the filler upon exposure to water, resulting in a higher level of cumulative Ca and P release for Renewal MI. For FUJI VII, the primary calcium phosphate component is Recaldent (CPP-ACP). The calcium-to-phosphorus ratio of ACP ranges from 1.2 to 2.2 [42], which may contribute to a reduction in water solubility and the release of elements for FUJI VII. The dissolution of reactive fillers may, however, leave voids on the surface, which could subsequently increase the wear or surface roughness of the materials [43, 44]. The limitation of the current study was that the release was only measured in deionised water. Future studies should examine their release in lactic acid [45] to mimic the assessment of the remineralising effect during an acidic challenge in high caries-risk patients.

In this study, the disc of materials was adapted to demineralised dentine. The objective was to primarily examine whether composite or GICs could encourage ion release to promote mineral precipitation on the demineralised dentine. However, precipitates were not clearly observed on the demineralised dentin surfaces. This may be attributed to insufficient ion release from the Renewal MI or GICs, which failed to achieve the saturation levels necessary for mineral precipitation at 2 weeks [46]. Another negative outcome might be due to our study’s limitation of using polymerised or fully set materials on the dentine surface. The disc specimens were cured in close contact with the light-curing unit, resulting in the development of highly rigid polymer networks. This may hinder the diffusion and release of ions from the specimen surface to the demineralised dentine [47, 48].

The previous studies suggested that SBF immersion should be extended to approximately 2–6 months to clearly detect the mineral precipitation on composites, which contained MCPM and polylysine concentrations comparable to those found in Renewal MI [14, 15]. The findings of the present study are also consistent with the previous study on GICs incorporating calcium phosphate compounds, such as hydroxyapatite or Ca-Si fillers [49]. The study reported no superiority *in vitro* remineralisation of demineralised dentine compared with a control after a 7-day immersion period, and the mineralising effects observed in the dentine may be primarily due to the effect of SBF [49]. An extended immersion duration may be required in the future study. This may allow further release of ions to aid in concentration and pH, which were supersaturated and suitable for the precipitation of mineral apatite [50].

The limitation of this study was the absence of an initial mineralisation profile of the specimen for comparison at the final time point. This should be addressed in future studies by using non-destructive analyses, such as ATR-FTIR [51] or X-ray diffraction analysis (XRD), to compare the mineral characteristics of specimens before and after treatment. Future research should also focus on applying uncured materials to surfaces and examining their remineralisation effects using confocal laser scanning microscopy (CLSM) with fluorescent markers or micro-computed tomography (micro-CT) imaging techniques to analyse the material interface.

## Conclusion

The novel resin composite (Renewal MI), containing MCPM and polylysine, showed a degree of conversion comparable to that of the commercial resin composite but lower than RMGIC. The strength values of the novel resin composite were similar to RMGIC and higher than those of conventional GIC. Renewal MI also promoted higher levels of Ca and P release compared to the conventional GIC added with calcium phosphate fillers (FUJI VII). This was expected to enhance its remineralisation of demineralised dentine.

## Data Availability

The data that support the findings of this study are available from the corresponding author upon reasonable request.
